# Low Measles Seropositivity in Vaccinated Children

**DOI:** 10.1001/jamanetworkopen.2025.29409

**Published:** 2025-08-27

**Authors:** Huy Quang Quach, Sara P. Jones, Iype Joseph, Alexandria J. Powell, Inna G. Ovsyannikova, Nathaniel D. Warner, Diane E. Grill, John B. Johnson, Remya Vasanthi Sasi, Archana Mohankumar Ajithakumari, Raji Prasad, Remya Reveendran, Jayalekshmi Devakikutty, Vishnu Vikraman Mohanakumari, Gregory A. Poland, M. Radhakrishna Pillai, Joshy Jacob, Richard B. Kennedy

**Affiliations:** 1 Mayo Clinic Vaccine Research Group, Department of Internal Medicine, Mayo Clinic, Rochester, Minnesota; 2 Pathogen Biology, BRIC-Rajiv Gandhi Centre for Biotechnology (BRIC-RGCB), Jagathy, Thiruvananthapuram 695014, Kerala, India; 3 Department of Quantitative Health Sciences, Mayo Clinic, Rochester, Minnesota; 4 Department of Microbiology and Immunology, Emory University School of Medicine, Atlanta, Georgia

## Abstract

**Importance:**

India’s goal of measles elimination remains unmet, as evidenced by significant recent outbreaks.

**Objective:**

To identify seroprevalence rates among vaccinated children and to examine demographic factors that influence antibody responses.

**Design, Setting, and Participants:**

This cross-sectional study included a highly vaccinated cohort of Indian children from the states of Kerala and Tamil Nadu between 2018 and 2023. Eligible children had received at least 2 doses of measles-containing vaccines; their mothers were recruited from various community settings, including local nursery and secondary schools, residential associations, and hospitals.

**Exposure:**

Measles-specific antibodies were quantified in serum samples.

**Main Outcomes and Measures:**

Measles-specific immunoglobulin G (IgG) and neutralizing antibodies were quantified in both children and mothers. Measles-specific IgM was also measured in children.

**Results:**

The total cohort comprised 684 children (median age, 9 years [range, 4-18 years]; 348 male [50.1%]) and 544 mothers. All children received at least 2 doses of measles-containing vaccines; 435 children (63.6%) received a third dose, and 7 (1.0%) received a fourth dose. Among the children, 621 (90.8%) had positive measles-specific IgG, and 623 (91.5%) had protective neutralizing antibodies titers, with a strong correlation between measles-specific IgG and neutralizing antibodies (*r* = 0.73; *P* < .001). Female children exhibited significantly higher titers of both measles-specific IgG and neutralizing antibodies compared with male children. While IgG and neutralizing antibody titers remained stable over time and were not associated with the number of vaccine doses in children, neutralizing antibody titers increased with age in mothers, likely due to repeated viral exposure. Notably, in 20 families with at least 2 children, differential measles-specific IgM profiles were observed between siblings despite high IgG and neutralizing antibody titers, suggesting ongoing breakthrough infections.

**Conclusions and Relevance:**

In this cross-sectional study, substantial measles immunity gaps were found despite high vaccine coverage with evidence of breakthrough infections, posing significant challenges to India’s measles elimination efforts. These results underscore the urgent need to strengthen India’s immunization program and investigate the mechanisms driving suboptimal responses to measles vaccination; without addressing these immune response deficiencies, achieving measles elimination through increased vaccine coverage alone may remain unattainable in India.

## Key Points

**Question:**

What is the measles-specific antibody seroprevalence among vaccinated children in southern India?

**Findings:**

In this cross-sectional study of 684 children and their 544 mothers from the states of Kerala and Tamil Nadu, 90.8% of vaccinated children had positive measles-specific immunoglobulin G (IgG) and 91.5% had protective neutralizing antibody titers. The presence of measles-specific IgM in 13% of vaccinated children and the elevated neutralizing antibody titers in mothers suggest ongoing virus circulation within the community.

**Meaning:**

These findings highlight immunity gaps in vaccinated children in India hindering measles elimination efforts, emphasizing the need to elucidate mechanisms driving suboptimal immune responses to measles vaccination.

## Teaser

This cohort study of children and mothers from 2 provinces in India examines rates of measles-specific antibody seropositivity among vaccinated children.

## Introduction

India’s goal to eliminate measles by 2020,^1^ later revised to 2023 due to the COVID-19 pandemic, remains unmet, with India remaining a major contributor to global measles cases.^2^ India conducted a mass vaccination campaign from 2017 to 2020, raising 2-dose measles vaccine coverage from 73% to over 90%.^3,4^ However, vaccine coverage does not accurately reflect population immunity, as suboptimal immune responses to vaccination have been reported.^5,6^ Quantitative antibody titers, which are linked to correlates of protection,^7^ may provide more reliable insights for policymaking. In this study, we evaluated measles antibody seroprevalence in 684 vaccinated children and 544 mothers from the states of Kerala and Tamil Nadu, India, and examined demographic factors associated with antibody responses. Our findings reveal substantial immunity gaps, even among fully vaccinated children, underscoring the need for intensified efforts to achieve measles elimination in India.

## Methods

### Ethics Statement

This study was approved by the Mayo Clinic institutional review board and the institutional human ethics committee of Rajiv Gandhi Centre for Biotechnology. Written informed consent was obtained from all participants. This study followed the Strengthening the Reporting of Observational Studies in Epidemiology (STROBE) reporting guideline for cross-sectional studies.

### Study Participants

This study involved children who had received at least 2 doses of measles-containing vaccines and their corresponding mothers from the states of Kerala and Tamil Nadu, India. Participants were recruited from diverse community settings, including nursery schools, secondary schools, residential associations, and hospitals during noninfectious illness visits.

### Quantification of Measles-Specific Antibodies

Measles-specific IgG (MEAG0330) and IgM (MEAM0330) levels were measured using commercial ELISA (enzyme-linked immunosorbent assay) kits (Novatec Immundiagnostica) following the manufacturer’s instructions. The IgG ELISA kit has an average intra-assay coefficient of variation (CV) of 6.87% and an inter-assay CV of 7.44%. It has a specificity of 100% (95% CI, 90.0%-100%) and a sensitivity of 97.0% (95% CI, 93.9%-98.8%). The IgM ELISA kit has an average intra-assay CV of 4.29%, an average interassay CV of 6.94%, with a specificity of 100% (95% CI, 98.7%-100%) and a sensitivity of 100% (95% CI, 91.2%-100%).

Measles-specific neutralizing antibodies against Edmonston strain were quantified using a fluorescence-based plaque reduction microneutralization assay, as detailed in our previous study.^8^ Briefly, serum samples were serially diluted 4-fold (1:4 to 1:4096, with 6 replicates per dilution) in reduced serum medium (Invitrogen Corporation) and mixed in equal volume with green fluorescent protein (GFP)-expressing measles virus (multiply of infection [MOI] = 0.5). Each 96-well microplate included a virus control without serum. After incubation at 37 °C with 5% CO_2_ for 1 hour, 50 μL of the serum-virus mixture was transferred to a new 96-well microplate containing Vero cells (2 × 10^4^ cells/well) in Dulbecco modified Eagle medium (DMEM) supplemented with 10% FBS and penicillin or streptomycin. The plates were incubated for 42 hours at 37 °C with 5% CO_2_. Fluorescent plaques were imaged and quantified using the ImageXpress Nano platform and the MetaXpress software (Molecular Devices). The 50% neutralizing dose was calculated using the Karber formula and converted to mIU/mL based on the *3rd International Standard Anti-Measles Serum* (NIBSC code No. 97/648).^8^ A neutralizing antibody titer of 120 mIU/mL or higher was considered protective.^7^ The assay had a CV of 5.7% and a detection limit of 15 mIU/mL in our laboratory.

### Statistical Analysis

Continuous variables are presented as median or mean, as appropriate. Differences in antibody titers between sexes were assessed using the Wilcoxon rank-sum test, while the Kruskal-Wallis test compared titers across vaccine dose groups. Associations between age and antibody titers were evaluated using Spearman correlation. A *P* < .05 was considered statistically significant. All analyses were conducted and figures were generated using RStudio version 2024.09.0 + 375 (Posit).

## Results

### Characteristics of Study Cohorts

This study included 684 children and 544 mothers from Kerala and Tamil Nadu, India (Table). The child cohort (336 female [49.1%]) had a mean (SD) age of 9.0 (3.0) years. All children received at least 2 doses of measles-containing vaccines (including 1 dose of MMR vaccine); 435 (63.6%) received a third dose, and 7 (1%) received a fourth dose (Table). The mothers had a mean (SD) age of 35.2 (5.2) years. Vaccination records were largely unavailable for the maternal cohort. Among the 63 mothers with vaccination records, 54 reported no measles vaccination, 6 received 1 dose, and 3 received 2 doses (Table).

**Table.** Demographic and Clinical Characteristics of Study Participants

Characteristics	Individuals, No. (%)^a^	
	Children (n = 684)	Mother (n = 544)
Sex		
Male	348 (50.9)	NA
Female	336 (49.1)	544 (100)
Age, y		
No. missing	NA	4
Mean (SD)	9.0 (3.0)	35.2 (5.2)
Median (IQR) [range]	9 (7-11) [4-18]	35 (31-39) [22-52]
No. of measles vaccines		
No. missing	0	481 (88.4)
None	0	54 (9.9)
1	0	6 (1.1)
2	242 (35.4)	3 (0.6)
3	435 (63.6)	NA
4	7 (1.0)	NA
Measles-specific neutralizing antibody, mIU/mL		
Missing data, No. (%)	3 (0.4)	3 (0.6)
Mean (SD)	872.8 (2384.2)	2777.78 (3851.72)
Median (IQR) [range]	422.4 (230.1-819.7) [4.4-48 350.2]	1421.1 (559.2-3629.3) [12.4-35 281.9]
Protective threshold of measles neutralizing antibody (120 mIU/mL), No.		
Above	623	513
Female	314	NR
Male	309	NR
Below	58	28
Female	21	NR
Male	37	NR
Measles IgG (U/mL)		
No. missing	0	0
Mean (SD)	21.5 (9.1)	28.7 (10.7)
Median (IQR) [range]	20.4 (14.6-26.8) [0.4-58.2]	29.0 (22.1-35.8) [1.9-64.6]
Measles IgG, inference		
Negative (<9 unit/mL)	32 (4.7)	24 (4.4)
Borderline (9-11 unit/mL)	31 (4.5)	5 (0.9)
Positive (>11 unit/mL)	621 (90.8)	515 (94.7)
Measles IgM, unit/mL		
No. missing	0	NA
Mean (SD)	7.7 (3.8)	NA
Median (IQR) [range]	6.8 (5.3-9.1) [1.2-45.2]	NA
Measles IgM (inference)		
Negative (<9 unit/mL, %)	505 (73.8)	NA
Borderline (9-11 unit/mL, %)	90 (13.2)	NA
Positive (>11 unit/mL, %)	89 (13.0)	NA

Abbreviations: IgG, immunoglobulin G; NA, not applicable; NR, not reported.

^a^ All children received at least 2 doses of measles-containing vaccines while vaccination record was largely unavailable for mothers. Measles IgM data were not available for the mother cohort.

### Seroprevalence of Measles-Specific Antibodies

Among 684 children, 621 (90.8%) were positive, 32 (4.7%) tested negative, 31 (4.5%) were borderline for measles-specific IgG (Table). For measles-specific IgM, 505 (73.8%) were negative, 90 (13.2%) were borderline, and 89 (13.0%) were positive, suggesting recent infection among a significant minority of vaccinated children (Table). Unfortunately, medical records confirming exposure or measles-related symptoms were unavailable. Measles-specific neutralizing antibody data were available for 681 children, with a median titer of 422.4 mIU/mL; 58 (8.5%) had neutralizing antibody titers below the protective threshold (120 mIU/mL),^7^ while 623 (91.5%) had protective titers (Table). Among the 58 children with sub-protective neutralizing antibody titers, 33 received 2 doses (10 female, 23 male), 25 received 3 doses (11 female, 14 male). None of the children who received 4 doses had neutralizing antibody levels below the protective threshold.

**Figure 1.**
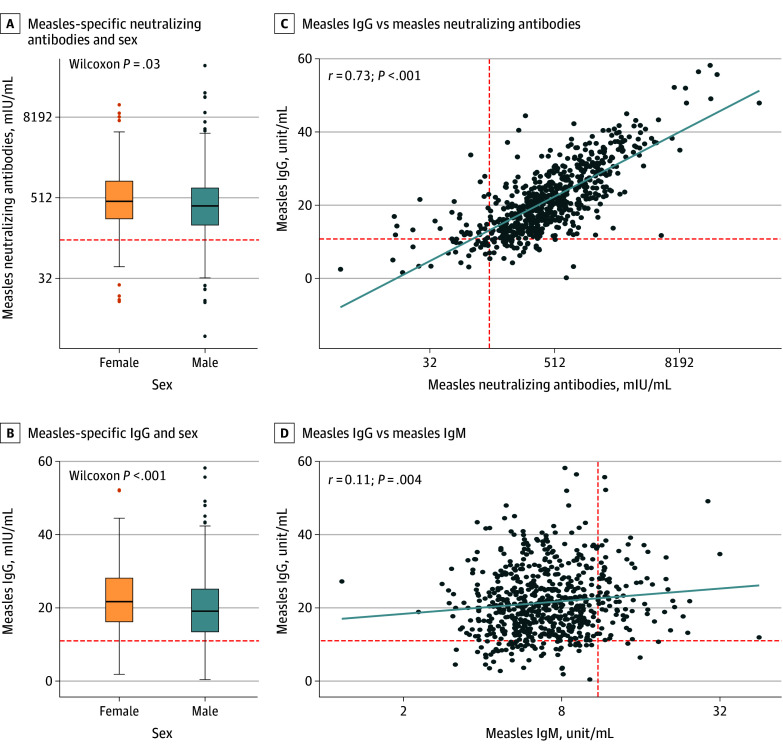
Association of Sex With Measles-Specific Antibodies in the Child Cohort A and C, The horizontal red dashed line and the vertical red dashed line represent the positivity threshold for measles-specific neutralizing antibodies (120 mIU/mL). B and C, The horizontal red line indicates the positivity threshold for measles-specific IgG (11 units/mL). D, The vertical red dashed line represents the positivity threshold for measles-specific immunoglobulin M (IgM) (11 units/mL).

**Figure 2.**
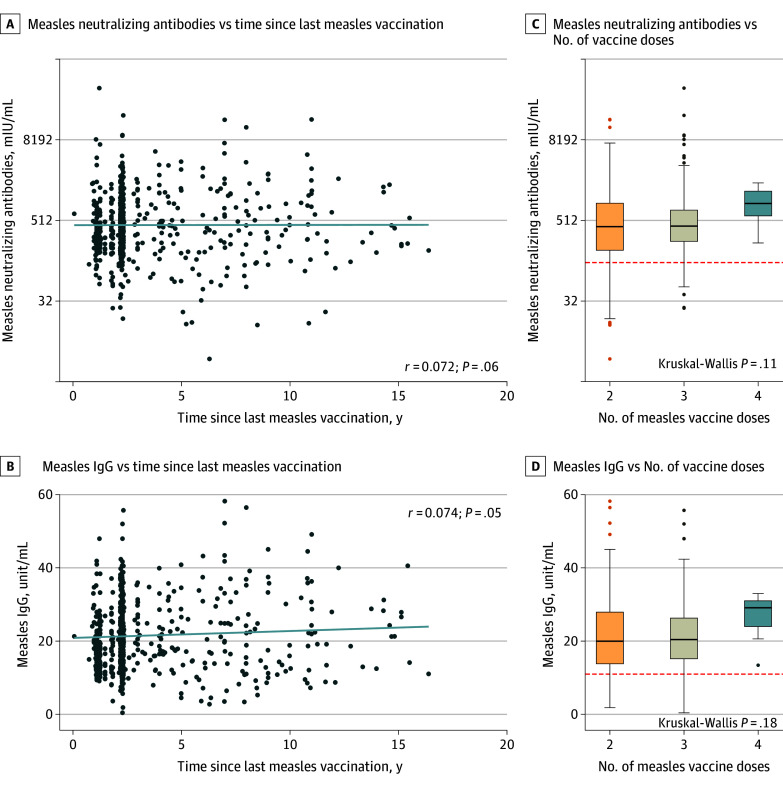
Time Since Vaccination and Number of Vaccine Dose on Measles-Specific Antibodies in the Child Cohort C, The horizontal red dashed line represents the positivity threshold for measle-specific neutralizing antibodies (120 mIU/mL). D, The horizontal red line represents the positivity threshold for measles-specific immunoglobulin G (IgG) (11 units/mL).

Female children had significantly higher neutralizing antibody (median [IQR] titer: female, 454 [249-907] mIU/mL vs male, 388 [200-716] mIU/mL; *P* = .03) and IgG (median [IQR] titer: female, 21.7 [16.2-28.1] unit/mL vs male, 19.1 [13.5-25.1] unit/mL; *P* < .001) titers than male children (Figure 1A and B). When stratified by the number of vaccine dose, this sex-based difference in neutralizing antibody titers remained significant among children who received 2 doses of measles-containing vaccines (242 children; median [IQR] titer: female, 449 [756] mIU/mL vs male, 337 [685] mIU/mL) (Table; eFigure in Supplement 1), but not among those who received 3 or 4 doses (442 children) (eFigure in Supplement 1). Measles-specific IgG titers strongly correlated with neutralizing antibody (*r* = 0.73, *P* < .001) (Figure 1C), but weakly with IgM (*r* = 0.11, *P* = .004) (Figure 1D). Neither neutralizing antibody nor IgG titers showed a significant correlation with time since the last measles vaccination, suggesting a minimal waning of immunity over time (Figure 2A-B). Similarly, there was no significant association between IgG titers and the number of vaccine doses received (Figure 2C-D).

**Figure 3.**
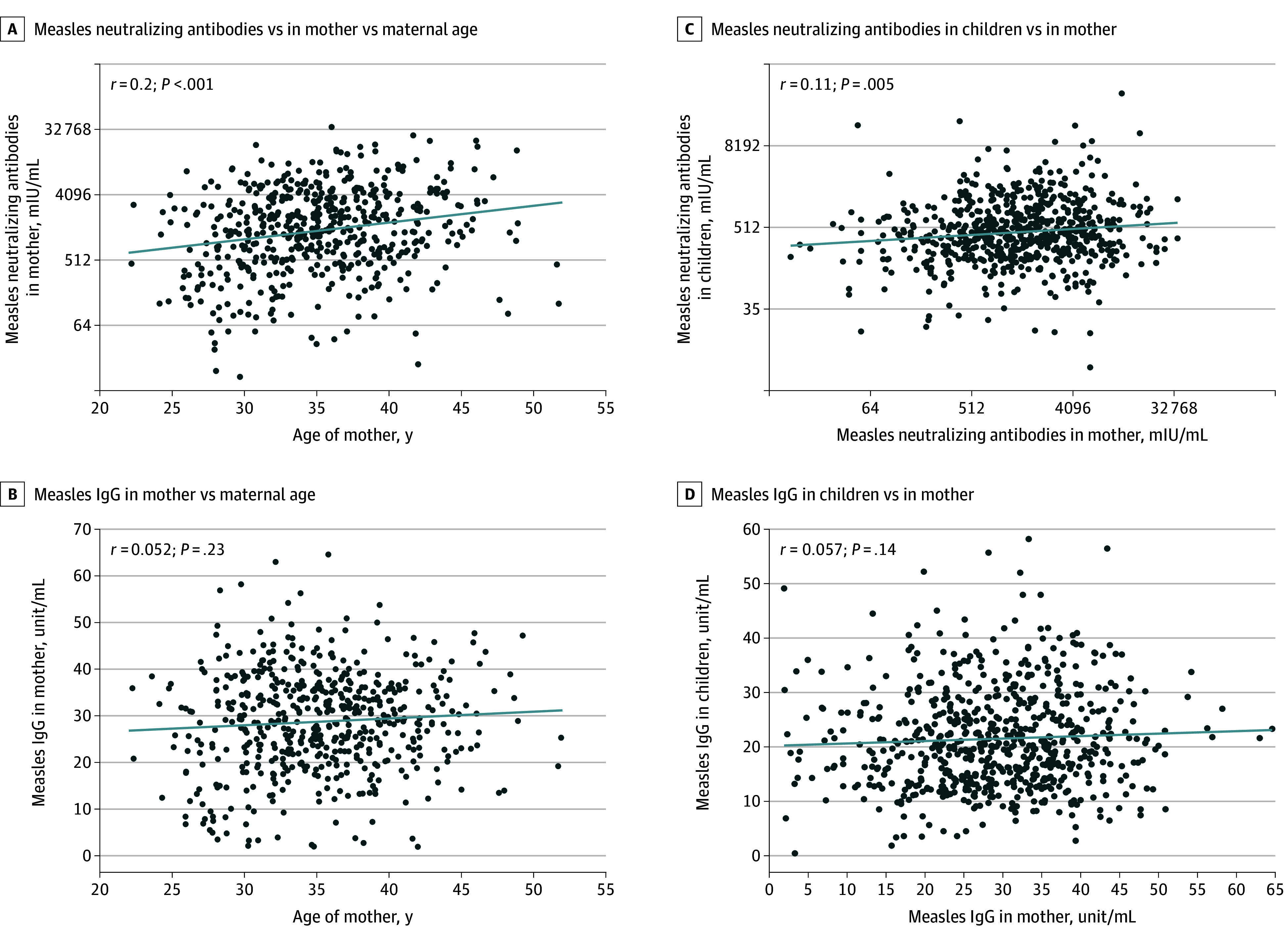
Correlations Between Measles-Specific Antibodies in Mothers and Their Children

Of 544 mothers, 515 (94.7%) tested positive, 5 (0.9%) were borderline, and 24 (4.4%) tested negative for measles-specific IgG (Table). Among the 541 mothers with neutralizing antibody data, the median neutralizing antibody titer was 1421.05 mIU/mL; 513 mothers (94.8%) had protective neutralizing antibody titers, while 28 (5.2%) fell below the threshold. In mothers, neutralizing antibody titers positively correlated with age (*r* = 0.20, *P* < .001) (Figure 3A), but IgG titers did not (Figure 3B). Additionally, maternal neutralizing antibody titers significantly correlated with their children’s neutralizing antibodies (*r* = 0.11, *P* = .005) (Figure 3C), although no correlation was seen for IgG (Figure 3D).

### Differential Measles-Specific IgM Profiles in 20 Families

**Figure 4.**
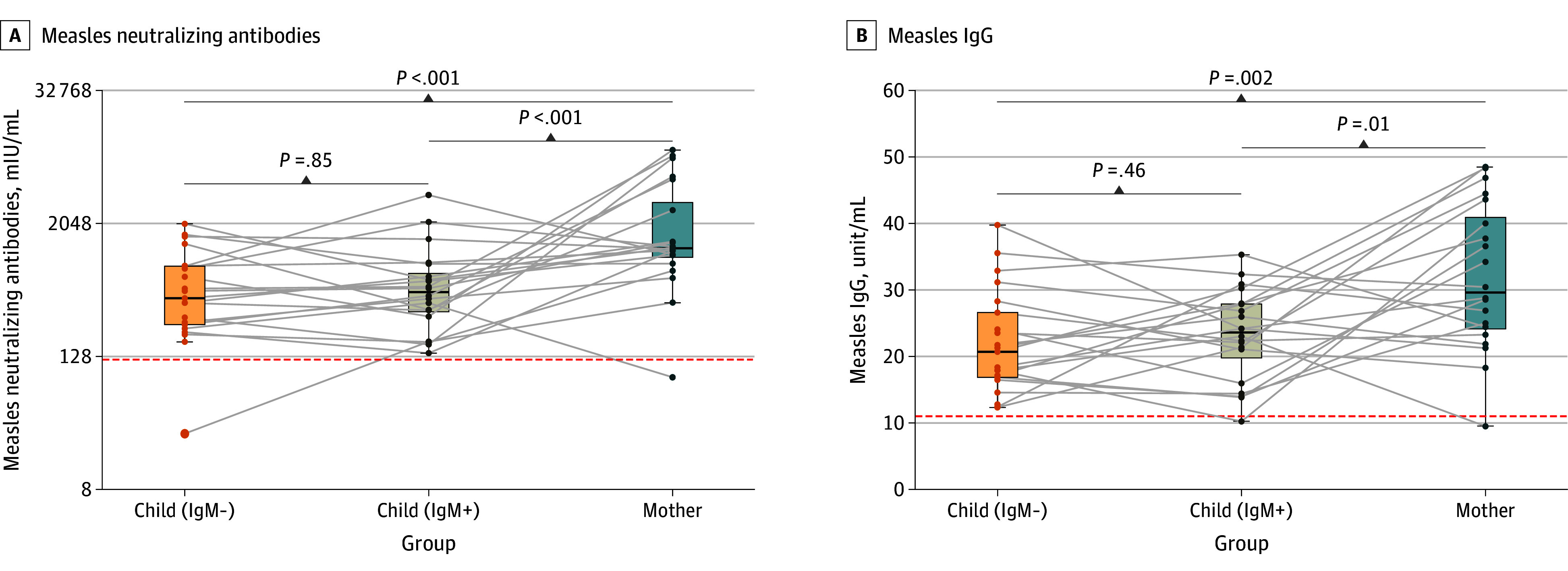
Differential Measles-Specific Immunoglobulin M (IgM) Profiles in Families With 2 or More Children In each of 20 families analyzed, 1 child tested negative while other siblings tested positive for measles-specific IgM. Despite this difference, IgM-negative and IgM-positive children had comparable titers of measles-specific nAb and measles-specific IgG. A and B, The red dashed lines in represent the positivity threshold for measles-specific neutralizing antibodies (120 mIU/mL) and measles-specific IgG (11 units/mL), respectively. The gray lines connect data points for children and mothers within the same household.

Among 149 mothers with at least 2 children, 20 families (41 children) had 1 child IgM-positive and their sibling IgM-negative, indicating differing recent measles exposure within the household (Figure 4). Of these 41 children, only 1 IgM-negative child had neutralizing antibody titers below the protective threshold (Figure 4A), and 1 IgM-positive child had IgG level below the positivity threshold (Figure 4B). While neutralizing antibody and IgG titers did not differ between IgM-negative and IgM-positive children, both groups had lower titers than their mothers (Figure 4).

## Discussion

Measles is one of the most contagious and deadly infectious diseases, yet it is highly preventable through vaccination, with 2 doses of the MMR vaccine providing approximately 97% efficacy in developed countries.^9^ Despite 100% vaccination coverage in our child cohort, where all children received at least 2 measles-containing vaccines, 63.6% received 3 doses, and 1% received 4, we observed a concerningly high rate of suboptimal immunity. Specifically, only 90.8% of children were positive for measles-specific IgG, and only 91.5% had protective neutralizing antibody titers (Table). These rates are lower than those typically reported in the US and Europe,^10,11^ and fall below the 95% threshold required for herd immunity,^12^ raising a serious concern about the adequacy of vaccine-induced protection in this population.

This concern is further exacerbated by 2024 survey data^13^ showing that 11.5% of 43 864 Indian children aged 24 to 35 months had not received any measles vaccination. Additionally, the detection of measles-specific IgM in 13% of children in our study, along with elevated neutralizing antibody titers in the mother cohort, suggests ongoing virus circulation within the community—a finding consistent with the continued reporting of significant measles cases in India.^1,14,15^ This concern extends beyond India, as importation remains a primary driver of measles outbreaks globally, including recent outbreaks in the US.^16^ India, therefore, faces a dual challenge: inadequate vaccine coverage and suboptimal immune responses even among fully vaccinated children. Closing these immunity gaps will require both strengthening immunization programs and investigating the underlying factors contributing to suboptimal vaccine-induced immune responses. Solutions to both issues are likely necessary to achieve and sustain measles elimination in India.

Consistent with previous studies,^8,17,18^ our child cohort demonstrated a clear association of sex with measles humoral immunity, with female children exhibiting significantly higher titers of measles-specific antibody titers compared with male children (Figure 1A-B). This observation aligns with a 2022 meta-analysis^19^ that reported higher measles incidence rates in male children compared with female children across various age groups over a period of 11 to 27 years from 7 countries. Given that measles-neutralizing antibodies are well-established correlates of protection against measles infection,^7^ these findings highlight the potential need for sex-based vaccination strategies. Male children, for instance, may benefit from enhanced measles vaccines, such as higher-dose or adjuvanted formulations, similar to the influenza vaccines recommended for older adults,^20^ or from additional booster doses.

Our data support this sex-based approach: among children with subprotective neutralizing antibody titers (58 children), 33 received 2 doses (10 female, 23 male), and 25 received 3 doses (11 female, 14 male); yet none of the children who received 4 doses had subprotective neutralizing antibody titers. Notably, this improvement appeared to be driven primarily by male children. Therefore, these findings underscore the potential of tailored vaccination strategies to reduce sex-based disparities in vaccine-induced immunity and improve overall vaccine effectiveness.

Given that measles is among the most contagious infectious disease with an infectivity approaching 100% and an estimated basic reproduction number of approximately 18 in susceptible individuals in close contact,^21^ the mechanisms driving the observed differences in the measles-specific IgM profiles among siblings in the 20 households remain unclear. Vaccine records showed no recent vaccination, suggesting that the IgM positivity likely reflects recent exposure to circulating measles virus. Considering the high transmissibility of measles, it is reasonable to assume that all household members were exposed. We initially hypothesized that IgM-positive children would have lower levels of measles-specific IgG and/or neutralizing antibodies. However, IgM-negative and IgM-positive siblings showed comparable titers of both measles-specific neutralizing antibodies and IgG. Several explanations might account for this finding: (1) IgM-negative children may have been exposed (and potentially infected) but had not yet mounted an IgM response; (2) protective cellular immune responses may have prevented active infection or disease in IgM-negative children; or (3) a true difference in IgG or neutralizing antibody titers exists, but the small sample size (20 families) limited our ability to detect statistical significance. Unfortunately, documentation of prior clinical measles infection or exposure was not available, preventing definitive conclusions.

Nevertheless, the comparable titers of measles-specific neutralizing antibodies and IgG across both groups suggest that cellular immunity may play a critical role in protection,^22,23^ particularly in the IgM-negative children. This highlights the need for follow-up studies to investigate the contribution of cellular immune responses to measles immunity, especially among children with suboptimal humoral responses. Additionally, our results clearly indicate that breakthrough infection can occur in measles-vaccinated children. This observation highlights the importance of reassessing the true level of protection conferred by current measles-containing vaccines in this population and support the need for strategies to enhance vaccine effectiveness.

Our findings underscore significant immunity gaps among vaccinated children that continue to hinder India’s progress toward measles elimination. A key insight from this study is that vaccine coverage does not necessarily equate to protective immunity. Therefore, herd immunity estimates should be based on the proportion of individuals with protective antibody levels, rather than simply on vaccine coverage metrics.^3,4^ Our findings also highlight the urgent need for further investigations into the factors that influence immune responses to measles vaccination in India. While variables such as cold chain maintenance,^24^ age at vaccination,^25^ nutritional status,^26^ underlying infections,^27^ genetic background,^28,29^ have been linked to suboptimal measles vaccination, their relative contributions in the Indian pediatric population remain poorly understood. Addressing these knowledge gaps will be essential to advancing measles elimination efforts in India—where the government has primarily focused on increasing 2-dose coverage. However, our data emphasize that immune responses remain insufficient even among fully vaccinated children, 64.6% of whom had received at least 3 doses.

### Limitations

Our study has several limitations. First, the lack of vaccination records for most of mothers prevented us from assessing the association between the number of vaccine dose and antibody titer in this group. Second, the absence of data on prior measles infections limited our ability to investigate the mechanisms underlying differential measles IgM profiles observed in 20 families as well as to distinguish between vaccine-induced from infection-induced humoral responses in our study cohorts. A better understanding of these mechanisms is crucial for preventing measles infection and, ultimately, for achieving measles elimination in India.

## Conclusions

This cross-sectional study reveals low rates of measles-specific neutralizing antibody and IgG in 684 vaccinated children in India, highlighting the urgent need to investigate mechanisms driving suboptimal immune responses to measles vaccination. Given measles’ extreme contagiousness, with an infectivity rate nearing 100% in close-contact setting, understanding these mechanisms is crucial for preventing measles transmission and achieving measles elimination in India.

## Notes

Supplement 1. **eFigure.** Significant Impact of Sex and Vaccine Dose Number on Measles-Specific Antibody Levels in Children

Supplement 2.
Data Sharing Statement
