# Supplementary material for: Low Measles Seropositivity in Vaccinated Children
Source: JAMA Netw Open. 2025 Aug 27;8(8):e2529409. doi: 10.1001/jamanetworkopen.2025.29409 (PMC12391999; doi:10.1001/jamanetworkopen.2025.29409)
Supplement: Supplement 1. — eFigure. Significant Impact of Sex and Vaccine Dose Number on Measles-Specific Antibody Levels in Children [file jamanetwopen-e2529409-s001.pdf]

Supplemental Online Content

Quach HQ, Jones SP, Joseph I, et al. Low measles seropositivity in vaccinated children. *JAMA Netw Open.* 2025;8(8):e2529409. doi:10.1001/jamanetworkopen.2025.29409

eFigure. Significant Impact of Sex and Vaccine Dose Number on Measles-Specific Antibody Levels in Children

This supplemental material has been provided by the authors to give readers additional information about their work.

© 2025 Quach HQ et al. *JAMA Network Open*.

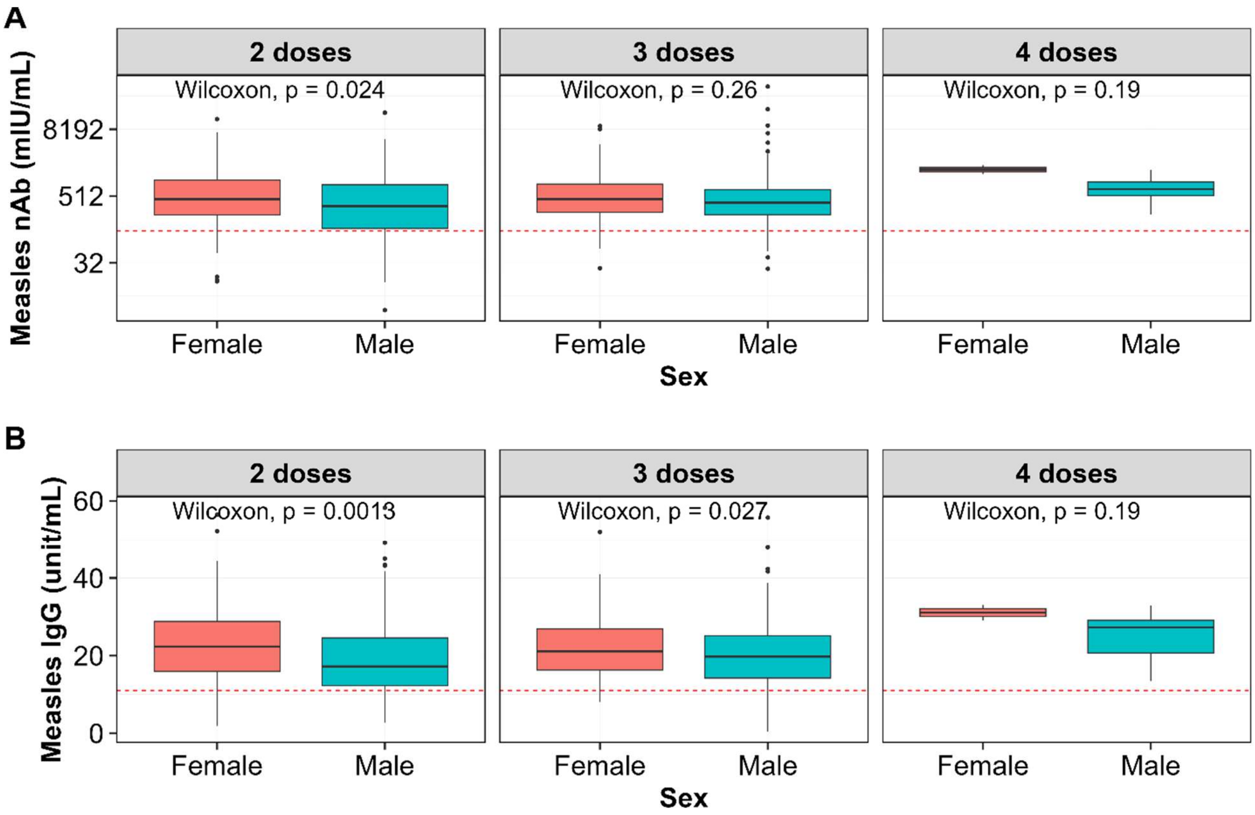

Supplementary Figure S1. Significant impact of sex and vaccine dose number on measles-specific antibody levels in children. Among children who received two doses of measles-containing vaccines, females had significantly higher titers of both measles-specific nAb (A) and IgG (B) compared to males. However, this sex-based difference was no longer significant for nAb titers among children received three or more dose (A), or for IgG levels among those who received four doses (B). In panel (A), the horizontal red dashed line in (A) represents the positivity threshold for measles-specific nAb (120 mIU/mL). In panel (B), the horizontal red lines in (B) marks the positivity threshold for measles-specific IgG (11 units/mL).

© 2025 Quach HQ et al. *JAMA Network Open*.
