# Supplementary material for: Low Measles Seropositivity in Vaccinated Children
Source: JAMA Netw Open. 2025 Aug 27;8(8):e2529409. doi: 10.1001/jamanetworkopen.2025.29409 (PMC12391999; doi:10.1001/jamanetworkopen.2025.29409)
Supplement: Supplement 2. — Data Sharing Statement [file jamanetwopen-e2529409-s002.pdf]

Data Sharing Statement

Quach. Low Measles Seropositivity in Vaccinated Children. *JAMA Netw Open*. Published August 27, 2025. doi:10.1001/jamanetworkopen.2025.29409

Data
Data available: Yes
Data types: Deidentified participant data
How to access data: Richard B. Kennedy, email: kennedy.rick@mayo.edu
When available: With publication

Supporting Documents
Document types: Statistical/analytic code
How to access documents: Richard B. Kennedy, email: kennedy.rick@mayo.edu
When available: With publication

Additional Information
Who can access the data: anyone requesting the data
Types of analyses: For research purpose only
Mechanisms of data availability: with investigator support
